# Heavy fermion quantum criticality at dilute carrier limit in CeNi_2−*δ*_(As_1−*x*_P_*x*_)_2_

**DOI:** 10.1038/s41598-019-48662-8

**Published:** 2019-08-23

**Authors:** Jian Chen, Zhen Wang, Yupeng Li, Chunmu Feng, Jianhui Dai, Zhu’an Xu, Qimiao Si

**Affiliations:** 1 Zhejiang Province Key Laboratory of Quantum Technology and Device, Department of Physics, Zhejiang University, Hangzhou, 310027 China; 2 Zhejiang University of Water Resources and Electric Power, Hangzhou, 310018 China; 3 Department of Physics, Hangzhou Normal University, Hangzhou, 310036 China; 4 Zhejiang California International NanoSystems Institute, Zhejiang University, Hangzhou, 310027 P. R. China; 5 Collaborative Innovation Centre of Advanced Microstructures, Nanjing University, Nanjing, 210093 P. R. China; 6 Department of Physics and Astronomy, Rice University, Houston, Texas 77005 USA

**Keywords:** Phase transitions and critical phenomena, Superconducting properties and materials

## Abstract

We study the quantum phase transitions in the nickel pnctides, CeNi_2−*δ*_(As_1−*x*_P_*x*_)_2_ (*δ* ≈ 0.07–0.22) polycrystalline samples. This series displays the distinct heavy fermion behavior in the rarely studied parameter regime of dilute carrier limit. We systematically investigate the magnetization, specific heat and electrical transport down to low temperatures. Upon increasing the P-content, the antiferromagnetic order of the Ce-4*f* moment is suppressed continuously and vanishes at *x*_*c*_ ~ 0.55. At this doping, the temperature dependences of the specific heat and longitudinal resistivity display non-Fermi liquid behavior. Both the residual resistivity *ρ*_0_ and the Sommerfeld coefficient *γ*_0_ are sharply peaked around *x*_*c*_. When the P-content reaches close to 100%, we observe a clear low-temperature crossover into the Fermi liquid regime. In contrast to what happens in the parent compound *x* = 0.0 as a function of pressure, we find a surprising result that the non-Fermi liquid behavior persists over a nonzero range of doping concentration, *x*_*c*_ < *x* < 0.9. In this doping range, at the lowest measured temperatures, the temperature dependence of the specific-heat coefficient is logarithmically divergent and that of the electrical resistivity is linear. We discuss the properties of CeNi_2−*δ*_(As_1−*x*_P_*x*_)_2_ in comparison with those of its 1111 counterpart, CeNi(As_1−*x*_P_*x*_)O. Our results indicate a non-Fermi liquid phase in the global phase diagram of heavy fermion metals.

## Introduction

As a result of a continuous phase transition at zero temperature, quantum criticality has been broadly discussed in connection with remarkable low temperature properties such as non-Fermi liquid (NFL) and unconventional superconductivity in a number of strongly correlated electron systems^1–3^. In Kondo lattice systems, quantum criticality is attributed to the interplay between the Ruderman-Kittel-Kasuya-Yosida (RKKY) interaction and the Kondo couplings. The RKKY interaction between the localized 4*f* electrons, mediated by hybridized conduction electrons, stabilizes the 4*f* moments so that the system tends to show magnetic order at low temperature. On the other hand, if the Kondo effect is dominant, the 4*f* moments are screened by conduction electrons so that the Fermi liquid behavior appears at low temperature. In the transition regime, the competition between the two types of interactions are apparent at temperatures below the coherence temperature, where the effect of the coupling between the local moments and conduction electrons sets in. The ground state due to the competition between the RKKY interaction and the Kondo effect can be generally understood by the Doniach phase diagram^4^. A magnetic quantum critical point(QCP) may arise in this phase diagram by varying the Kondo coupling or density of charge carriers at the Fermi energy, although its existence is always complicated in realistic materials. The energy scale for the Kondo effect, Kondo temperature *T*_*K*_, depends on the *c*-*f* hybridization and the density of states at the Fermi energy, and thus can be tuned by non-thermal parameters such as external pressure, chemical doping or applied magnetic field, etc. This allows to probe a possible QCP by identifying the associated NFL behavior in its vicinity^5,6^.

In this context, what has been little explored is the limit where the charge carriers are very dilute. In this regime, one may be concerned that the number of conduction electrons is insufficient to screen all the local moments completely. This is the so-called Nozières’ exhaustion problem^7^. The idea behind this problem is that the collective Kondo screening can still take place in the limit of dilute conduction electrons but with delayed development of coherent paramagnetic Kondo singlet state. This scenario has recently been evidenced in the cerium based nickle arsenide, CeNi_2−*δ*_As_2_^8^, where the unconventional quantum criticality has been revealed by tuning the physical pressure^9^. More generally, the dilute carrier concentration can lead to new types of underlying heavy fermion state and the associated quantum phase transitions^10^. In order to explore this physics, it is desirable to have as large a coherence temperature as possible. Because the coherence temperature is increased by the isovalent P for As substitution^11^, we have been motivated to study the antiferromagnetic (AFM) quantum phase transition by the effect of chemical pressure in CeNi_2−*δ*_(As_1−*x*_P_*x*_)_2_.

CeNi_2−*δ*_As_2_, with *δ* ≈ 0.28, crystallizing in the well-known ThCr_2_Si_2_-type structure ($I4/mmm$, No.139), was found to be a Kondo compound, with AFM transition temperature *T*_*N*_ = 4.8 K^8^. When the magnetic field was applied along the *c*-axis, the well localized moments of Ce 4*f* electrons undergo a spin-flip metamagnetic transition (MMT), from an AFM to a polarized paramagnetic (PM) ground state. Moderately strong electronic correlation is indicated by an enhanced electronic Sommerfeld coefficient *γ*_0_ ≈ 65 mJ/mol · K^2^ and a Kondo temperature *T*_*K*_ ~ 4 K, rendering CeNi_2−*δ*_As_2_ as a new system for unraveling the competition between the RKKY interaction and Kondo effect. In fact, two possible QCPs in CeNi_2−*δ*_As_2_ were reported previously^8,9^: one is induced by hydrostatic pressure at *p*_*c*_ = 2.7 GPa and the other by magnetic field at *B*_*c*_ = 2.8 T, the latter likely being a first-order quantum critical end point. In particular, due to the possible Ni vacancies in this compound under the ambient pressure, the density of charge carriers is expected to be small.

On the other hand, the isostructural compound CeNi_2−*δ*_P_2_ was found to be a nonmagnetic, intermediate valence Kondo lattice metal^12^. CeNi_2−*δ*_As_2_ and CeNi_2−*δ*_P_2_ are thus located at the opposite sides of the QCP in the Doniach phase diagram. Therefore, it is natural to consider the P substitution for As in the Ni-As layer of CeNi_2−*δ*_As_2_. As P has a smaller ionic radius than As, this isovalent substitution is expected to introduce chemical pressure and could shift the system from the AFM to the non-magnetic ground states. If the Ni vacancies do not change very much under the As/P substitution, we anticipate that CeNi_2−*δ*_(As_1−*x*_P_*x*_)_2_ should be a suitable system to investigate whether there is a QCP in a Kondo lattice with a low carrier density.

In this paper, we report a comprehensive study on the Kondo compounds CeNi_2−*δ*_(As_1−*x*_P_*x*_)_2_ (*δ* ≈ 0.07–0.22) using chemical doping concentration *x* as a tuning parameter. A *T*-*x* phase diagram is then determined for $0\le x\le 1$, and evidence for the low carrier density is found for $0.1\le x\le 0.7$. From the results of magnetic susceptibility $\chi (T)$, specific heat coefficient $C(T)/T$ and electrical resistivity ${\rho }_{xx}(T)$, we find that the AFM order is suppressed continuously and disappears at *x*_*c*_ ≈ 0.55. Around this QCP, the NFL behavior is exhibited in ${\rho }_{xx}(T)$ and $C(T)/T$, which indicates a divergent effective mass. We find the surprising result that the NFL behavior persists over a nonzero range of doping concentration; the Fermi liquid behavior is not recovered until $x\ge 0.9$. In the doping range of $0.55 < x < 0.9$, NFL behavior is observed at low temperatures: the electrical resistivity is linear in temperature and the specific-heat coefficient is logarithmically divergent. We discuss the properties of CeNi_2−*δ*_(As_1−*x*_P_*x*_)_2_ together with those of its 1111 counterpart, CeNi(As_1−*x*_P_*x*_)O, and draw implications for the global phase diagram of the heavy fermion metals.

## Results and Discussion

### Sample characterizations

**Figure 1:**
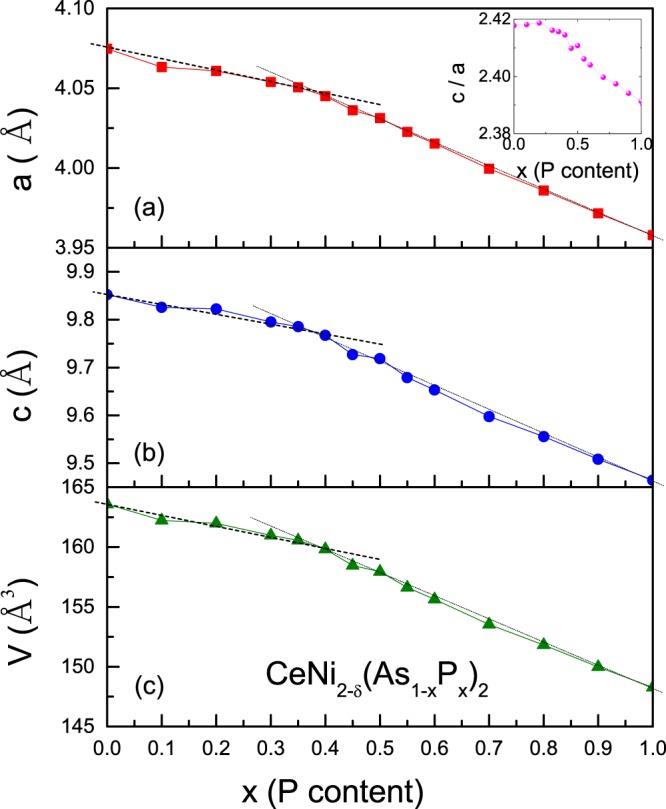
Lattice parameters *a*, *c* and unit-cell volume *V* as a function of the P content *x* in (**a**–**c**). The dashed and dotted lines are linear fit for *x* = 0.0–0.4 and 0.5–1.0, respectively. Inset to (**a**) shows the ratio of *c*/*a*.

The room temperature XRD measurements were performed on several CeNi_2−*δ*_(As_1−*x*_P_*x*_)_2_ samples. Almost all patterns can be well indexed with the ThCr_2_Si_2_-type body centered tetragonal structure with the space group $I4/mmm$ (No. 139), except for a slight NiAs/Ni_2_P non-magnetic impurity in samples with low/high P-concentration (data not shown). The peaks shift to higher degree with increasing P-concentration, consistent with the fact that the ion radius of P is smaller than that of As. The lattice parameters *a*, *c* and the unit cell volume *V*, which are estimated by the least square method using at least 25 reflections, are plotted in Fig. 1 as a function of the P content *x*. Two distinct, linear regimes with different slopes are clearly discernible. All the lattice parameters for *x* ≥ 0.5 decrease faster than those with lower concentration. This is an indication of intermediate valence of cerium, since the ion radius of Ce^4+^ is smaller than that of Ce^3+^. This is confirmed by the previous report^12^ which shows that the Ce ion in CeNi_2−*δ*_P_2_ is in the intermediate valence state.

We also performed Rietveld refinement^13^ on the XRD data of the parent compounds CeNi_2−*δ*_As_2_ and CeNi_2−*δ*_P_2_ (data not shown). The lattice parameters *a* and *c* are 4.0745(1) *Å* (3.95789(4) *Å*) and 9.8514(3) *Å* (9.4650(1) *Å*) for the As- (P-) compound, respectively, in agreement with the previously reported values^8,12^. The derived *c*/*a* ratio collapses from 2.418 to 2.391 (seen from inset to Fig. 1(a)). This evidences an increased *c*-*f* hybridization or Kondo effect which will be discussed latter. The derived occupation of Ni site is 0.78 and 0.93 for As- and P-end compound, respectively, confirmed by the EDS analysis. Therefore, the number of Ni atom is obtained as 1-*δ*, where  *δ* ≈ 0.07–0.22 for this series compounds. From the EDS measurements, the molar ratio of Ce:Ni in all the series compounds is about 1:(1.71 ± 0.15), while the ratio of P to As is almost equal to the nominal value. So the Ni vacancies exist in both end compounds. Moreover, the sum of P and As contents is slightly higher than the nominal value. We note that a similar observation was reported in a number of other arsenic compounds^14^.

### Magnetic susceptibility and Hall resistivity

**Figure 2:**
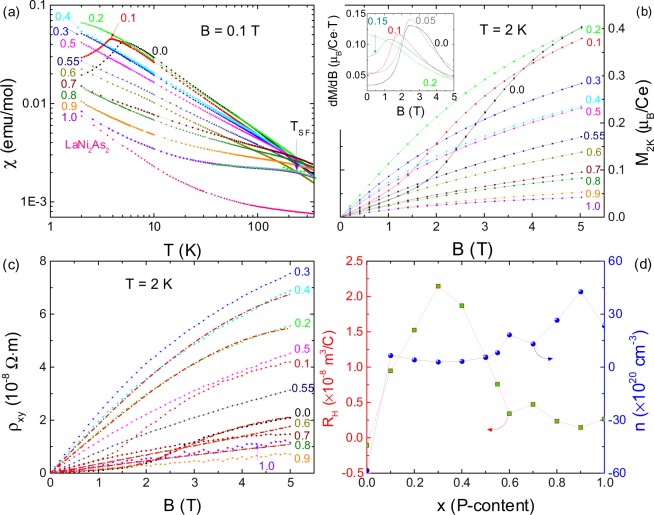
$\chi (T)$ (**a**) and *M*_2*K*_(*B*) (**b**) of CeNi_2−*δ*_(As_1−*x*_P_*x*_)_2_ for the selected concentrations. (**c**) Magnetic field dependence of the Hall resistivity, ${\rho }_{xy}(B)$, measured at *T* = 2 K. The red dashed lines present the fitted curves for selected samples (*x* = 0.0, 0.2, 0.4, 0.6 and 0.8) using Eq. 1 (see the text). (**d**) The *x* evolution of the Hall coefficient *R*_*H*_ and the carrier density *n* on the left and right panel, respectively. Inset to (**b**) presents d*M*/d*B* versus *B* at *T* = 2 K for *x* = 0.0–0.2.

**Figure 3:**
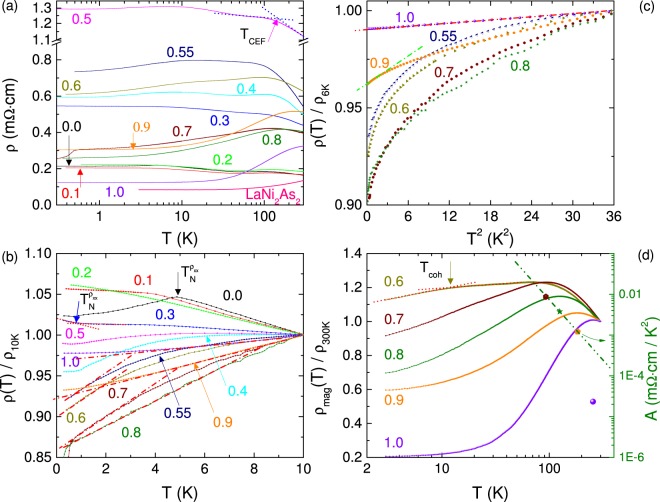
(**a**) Temperature dependence of the electrical resistivity ${\rho }_{xx}(T)$ for CeNi_2−*δ*_(As_1−*x*_P_*x*_)_2_ series as well as LaNi_2_As_2_ in a log-log scale. The normalized resistivity for the selected concentrations is shown as ${\rho }_{xx}$/*ρ*_1__0__*K*_ versus *T* below 10 K in (**b**) and ${\rho }_{xx}$/${\rho }_{6K}$ versus *T*^2^ below 6 K in (**c**), respectively. The dashed-dotted lines are linear fit to ${\rho }_{xx}$. The normalized resistivity ${\rho }_{xx}(T)$/${\rho }_{300K}$ at *T* = 2–400 K on the left axis of (**d**) for *x* = 0.6–1.0 samples. The log-log plot of *A* coefficient versus *T*_*coh*_ on the right axis of (**d**). The dashed-dotted-dotted line indicates $A\propto {({T}_{coh})}^{2}$. *T*_*CEF*_ (*T*_*coh*_) is shown by an arrow for *x* = 0.5 sample in (**a**) (*x* = 0.6 in (**d**)).

Figure 2(a) presents the temperature dependence of the molar magnetic susceptibility $\chi$ for eleven CeNi_2−*δ*_(As_1−*x*_P_*x*_)_2_ samples of different *x* measured at *B* = 0.1 T on a logarithmic plot. The magnetic susceptibility of isostructural nonmagnetic compound LaNi_2_As_2_ is shown for comparison. Several features are clearly revealed: (i) A kink at ${T}_{N}^{\chi }=4.8\,{\rm{K}}$, the characteristic of a second-order AFM transition, is pronounced for CeNi_2−*δ*_As_2_. As the amount of P is increased, the AFM transition shifts to lower temperature and falls below our base temperature for *x* ≥ 0.2. (ii) The temperature dependence of magnetic susceptibility, which is strong for the As-end compound, becomes rather weak with increasing *x*. Besides, the isothermal magnetization measured at *T* = 2 K decreases almost monotonously with increasing *x* as shown in Fig. 2(b), also suggesting the screening of Ce local moments, or delocalization of the *f* electrons. To interpret this tendency qualitatively, the $\chi (T)$ curves at high temperature are fitted with a modified Curie-Weiss law, $\chi ={\chi }_{0}+C/(T-\theta )$. Here ${\chi }_{0}$ is a temperature independent susceptibility from the core diamagnetism, the van Vleck and Pauli paramagnetism, *C* is the Curie constant and *θ* is the Weiss temperature. The effective moment *μ*_*eff*_, which could be derived from the Curie constant, is then estimated to be around 2.26 *μ*_*B*_/Ce, showing a very weak variation with *x* for *x* < 0.5. Here we recall that usually the 3*d* electrons of Ni ions do not show magnetism in the nickel based pnictides^8,15^, so the magnetic moments in CeNi_2−*δ*_(As_1−*x*_P_*x*_)_2_ should come from the Ce 4*f*^1^-electrons. The observed value of the effect moment is close to but slightly smaller than that of the free Ce^3+^ ion, 2.54 *μ*_*B*_. The slight reduction of the Ce moments is usually ascribed to the crystalline electric field (CEF) effect^8^. With increasing P content, *μ*_*eff*_ decreases to 1.88 *μ*_*B*_/Ce for *x* = 0.9, confirming the enhanced itineracy of 4*f*-electrons due to the strengthened coherent Kondo screening. (iii) As P concentration is increased to *x* = 0.7, a broad hump in susceptibility appears around 100 K, corresponding to the so-called spin fluctuation temperature *T*_*SF*_^1^. The position of this hump shifts to higher temperature and becomes more apparent as *x* increases. In CeNi_2−*δ*_P_2_, the Curie-Weiss law mentioned above is abided for *T* < 150 K, with a much reduced value of *μ*_*eff*_ = 0.34 *μ*_*B*_/Ce, while this law is violated in a high temperature range of *T* = 200–350 K. These results reveal the intermediate valence behavior for the compounds with $x\gtrsim 0.7$. Moreover, the characteristic temperatures *T*_*SF*_ and *T*_*coh*_, where $\chi (T)$ and ${\rho }_{mag}(T)$ exhibit respective local maxima, are located at almost the same temperature as shown by arrows in Figs 2(a) and 3(d). This observation favors the interpretation of *T*_*coh*_ in terms of spin-scattering mechanism in the intermediate valence state^1,16^.

The isothermal magnetizations *M*(*B*) for CeNi_2−*δ*_(As_1−*x*_P_*x*_)_2_, measured at *T* = 2 K in a *B*-sweep mode, is shown in Fig. 2(b). For the parent compound CeNi_2−*δ*_As_2_, *M*(*B*) displays a step-like behavior around 2.3 T, which is ascribed to a field-induced MMT^8^. In order to determine the MMT field *B*_*m*_ exactly, the derivative of magnetic field for the magnetization, d*M*/d*B*, is calculated as shown in the inset to Fig. 2(b). *B*_*m*_ is thus obtained at the peak of d*M*(*B*)/d*B*. As *x* is increased, *B*_*m*_ shifts from 2.3 T for *x* = 0.0 to a lower field of 1.21 T for *x* = 0.15 and is indiscernible for *x* ≥ 0.2 in the temperature limit of our measurement. It’s noted that the corresponding hysteresis around *B*_*m*_ of the first-order nature is indiscernible. This is probably because the magnetic correlations among cerium moments are strongly anisotropic; thus, the hysteresis detected only for $B\parallel c$ of single crystalline samples in ref. ^8^ is smeared out in the polycrystalline samples studied here.

We also measured the transverse Hall resistivity at *T* = 2 K as a function of the applied magnetic field ${\rho }_{xy}(B)$ as shown in Fig. 2(c). Interestingly, the field dependence of ${\rho }_{xy}$ resembles the magnetization, *M*, with the following features: (i) the nonlinearity in the full magnetic field range of *B* = 0–5 T for the whole system; (ii) a pronounced step-like increase at *B*_*m*_ = 2.3 T for dopants with *x* ≤ 0.1 without obvious hysteresis. The critical field *B*_*m*_ for the spin-flip MMT shifts to lower fields as the P-concentration is increased. It is noteworthy that the evolution of the magnetic field vs. P-doping concentration is remarkably similar to that of *B* − *p* relationship drawn in ref. ^9^.

With these observations, we fit the measured results for ${\rho }_{xy}(B)$ by the following expression:

1 $${\rho }_{xy}(B)={R}_{H}B+{R}_{S}{\mu }_{0}M(B).$$

The first term represents the normal Hall effect originated from the Lorentz force, while the second term represents the anomalous Hall effect (AHE) originated presumably from the magnetic fluctuations of the localized Ce 4*f*-spins, the skew scattering^17^. The derived Hall coefficient *R*_*H*_ and the carrier density *n*, using a simple single band model according to the relationship of ${R}_{H}\propto 1/n$, are presented in Fig. 2(d) for various *x*. The sign of *R*_*H*_ (*n*) changes from negative for *x* = 0.0 to positive for the rest of this family, while the anomalous Hall contribution *R*_*s*_ is always positive. In particular, the magnitude of *n* is quite small, showing a significant reduction in the range of 0.1 ≤ *x* ≤ 0.7, with a slight increase in the compounds on the P-rich side. The lowest magnitude is about one order smaller than about 30 × 10^20^ cm^−3^ for the end compound *x* = 1.0. This is consistent with the presence of the Ni vacancies mentioned previously. As we will show later, the low carrier density due to the Ni vacancies may account for the semimetal behavior in the As-rich compounds, and the slight increase of *n* in the P-rich side compounds may also explain the crossover from semimetal-like to metallic behavior at low temperature. It is, therefore, speculated that P-doping has both shifted the Fermi level and changed the dominant carrier from electron to hole. The reason that the electron carrier changes to hole between *x* = 0 and *x* = 0.1 remains unclear from our measurements. This is certainly an interesting issue which calls for further experiments down to much lower temperatures as well as theoretical study on the Fermi surface topology. The Hall resistivity data for CeNi_2−*δ*_As_2_ at different temperatures are also fitted using the above equation 1. *R*_*H*_ increases to a broad maximum around *T*_*coh*_ before decreasing sharply to a small value in the low temperature coherent-band regime (data not shown). This behavior is reminiscent of CeAl_3_, CeRu_2_Si_2_, and many other heavy fermion compounds^18^.

### Specific heat

**Figure 4:**
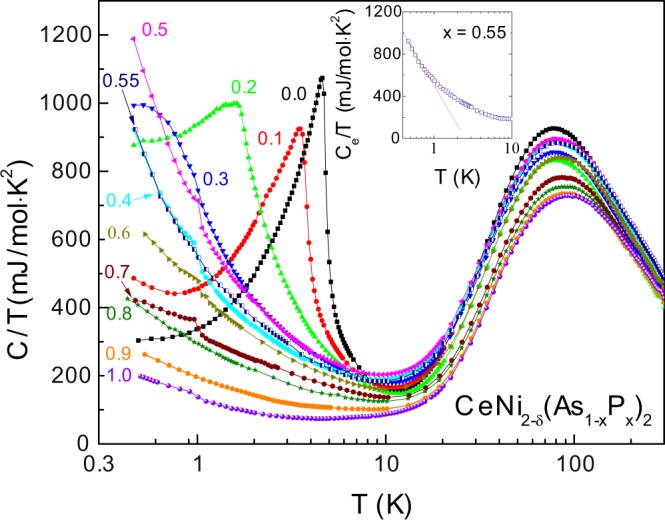
Temperature dependence of specific heat divided by temperature, *C*/*T*, for CeNi_2−*δ*_(As_1−*x*_P_*x*_)_2_. Inset displays the electronic contribution *C*_*e*_(*T*)/*T* for *x* = 0.55 at *T* = 0.4–10 K. The dashed line is a linear fit.

The specific heat coefficient of CeNi_2−*δ*_(As_1−*x*_P_*x*_)_2_, $C(T)/T$, is plotted in the main panel of Fig. 4 in semi-logarithm scale. A prominent feature for the As-rich compounds is the *λ*-type kink, typical of a second-order AFM transition. This peak is most pronounced in the parent compound, exhibited at 4.8 K in consistent with the previously reported value^8^. The magnetic transition temperature, ${T}_{N}^{C}$, can then be determined from the derivative of $C(T)/T$ at the sharp kink. For *x* = 0.0–0.4, the peak broadens and moves to lower temperature with increasing the P-content *x*. For *x* = 0.5, $C(T)/T$ increases drastically down to our base temperature while the derivative curve still drops down sharply. This is a characteristic feature of quantum criticality, implying a zero temperature QCP near $x=0.5$. Moreover, the electronic contribution ${C}_{e}(T)/T$ at *x* = 0.55, derived by subtracting the phonon term $\beta {T}^{2}$, increases in a logarithmic scale below ~1 K, shown in the inset to Fig. 4. This typical NFL behavior provides strong evidence for a divergent quasiparticle mass near the QCP. Far away from the critical point, the divergent tendency is suppressed to some extent, and finally, $C(T)/T$ tends to saturate at low temperature for *x* = 1.0, indicating the recovery of the paramagnetic Fermi liquid state. Note that there is another anomalous peak in $C(T)/T$ around 1 K for several samples. This anomaly probably comes from tiny amounts of unknown impurity phase, since its position does not change with P doping and nothing is observed from electrical resistivity curves over this temperature range. The upturn below 0.7 K in the *x* = 0.1 sample may be due to a nuclear quadrupolar Schottky anomaly arising mainly from the As atom.

**Figure 5:**
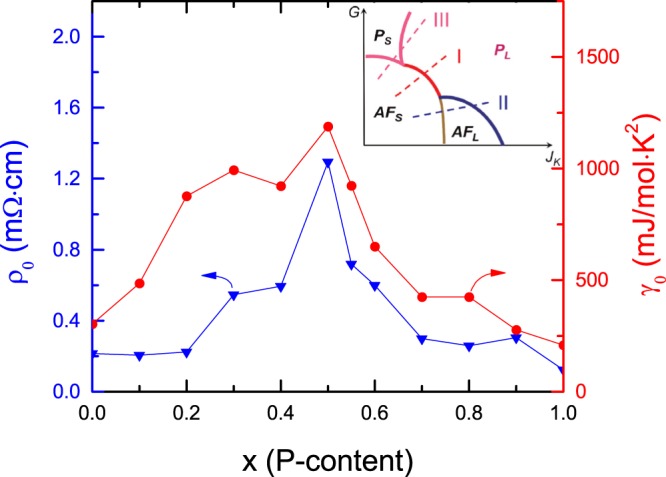
The evolution of residual resistivity ${\rho }_{0}$ and electronic Sommerfeld coefficient *γ*_0_ versus *x* on the left and right axis, respectively. Inset presents the global phase diagram for heavy fermion systems^36^. Here we actually used the resistivity and Sommerfeld coefficient values at *T* = 0.5 and 0.45 K as ${\rho }_{0}$ and *γ*_0_, respectively.

In order to characterize the variation of the Kondo coupling, or more precisely the hybridization between Ce-4*f* and the conduction electrons upon P doping, we also consider the variation of the Sommerfeld coefficient *γ*_0_ with increasing *x*. *γ*_0_ is extracted from $C/T$ data down to the base temperature *T* = 0.45 K since a divergent scattering is driven mainly by strong spin fluctuations around the QCP; besides, Kondo effect is expected to dominate over the RKKY interaction for *x* ≥ 0.6 samples. In Fig. 5, both Sommerfeld coefficient *γ*_0_ and the residual resistivity ${\rho }_{0}$ (stated below) show significant peaks at *x* = 0.5, in further support of a QCP near this doping level. We could expect that the peaks in *γ*_0_ and ${\rho }_{0}$ will be more divergent as temperature is further reduced forwards zero.

### Longitudinal electrical resistivity

The electrical resistivity ${\rho }_{xx}(T)$ in the full-*T* range and the normalized resistivity ${\rho }_{xx}(T)$/${\rho }_{10K}$ below 10 K of CeNi_2−*δ*_(As_1−*x*_P_*x*_)_2_ are shown in Fig. 3(a,b), respectively. For the parent compound CeNi_2−*δ*_As_2_, the AFM ordering phase is entered below ${T}_{N}^{{\rho }_{xx}}=4.88\,{\rm{K}}$, directly visible by the pronounced cusp in ${\rho }_{xx}(T)$. A negative logarithmic slop can be seen above ${T}_{N}^{{\rho }_{xx}}$ up to 40 K, indicating the Kondo effect. A broad hump develops at higher temperature *T*_*CEF*_ = 105 K which is due to the Kondo scattering on the excited CEF levels. When the P content increases, ${T}_{N}^{{\rho }_{xx}}$ shifts to lower temperature, as reflected by an increasing rounding in ${\rho }_{xx}(T)$. For *x* ≥ 0.2, the increase in ${\rho }_{xx}(T)$, namely an inflection, determined by the intercrossing of two dotted lines (arrow in Fig. 3(b)), signals the onset of AFM ordering. This inflection has been observed in CeCu_6−*x*_Au_*x*_ and many other Kondo lattice systems below *T*_*N*_^19^. Note that ${T}_{N}^{{\rho }_{xx}}$ is further suppressed to 0.45 K for *x* = 0.5 and invisible for *x* ≥ 0.55 in our measurement limit.

A remarkable feature for 0.0 ≤ *x* ≤ 0.5 is that ${\rho }_{xx}(T)$ increases slowly with decreasing temperature, showing the semimetal behavior at very low temperatures. While for *x* ≥ 0.6, the metallic behavior is exhibited below *T*_*coh*_, determined as the local maxima shown in Fig. 3(d). For $T < {T}_{coh}$, ${\rho }_{xx}(T)$ decreases monotonically with decreasing temperature, indicating the regime for the coherent Kondo screening. Note that in the P-rich case, the enhancement of electrical conductivity is clearly reflected by the decreased residual resistivity and increased carrier density *n*, shown in Figs 2(d) and 5, respectively, for higher doping concentrations. All these manifest the enhanced itineracy of Ce 4*f*-electrons with increasing *x* in the P-rich case. Moreover, a nearly linear temperature dependence in ${\rho }_{xx}(T)$ with varying slopes could be observed in the low-temperature regime for *x* = 0.55–0.8. Such linear resistivity behavior could be also extracted for *x* ≤ 0.9 over a limited range of *T* as shown by the dashed-dotted lines in Fig. 3(b). The lower end of that temperature range, denoted by *T*_*FL*_, signals the onset of the Fermi liquid state. The estimated *T*_*FL*_ increases from 0.95 K for *x* = 0.9 to 5.5 K for *x* = 1.0. Therefore, we further fit the low temperature resistivity in terms of a power law ${\rho }_{xx}(T)={\rho }_{0}+A{T}^{\alpha }$, for 0.5 K ≤ *T* ≤ 3 K. Such fitting is carried out only for *x* ≥ 0.55 because of the semimetal behavior when *x* ≤ 0.5. As P content is increased, the coefficient *A* is strongly decreased, and *α* varies from 0.67 for *x* = 0.55 to 2.41 for *x* = 1.0. The residual resistivity ${\rho }_{0}$, determined at *T* = 0.5 K, is plotted on the left panel of Fig. 5 as a function of *x*. The maximum value of ~1.3 mΩ · cm for *x* = 0.5 indicates strong quantum fluctuations near the critical point^20^, although high density of voids and/or microcracks can not be fully neglected. The residual resistivity ${\rho }_{0}$ for the As- and P-end compounds is likely due to the Ni-deficiency and/or the relatively low carrier density *n*. Note that the magnitude of ${\rho }_{0}$ of these Ni-based pnictides, falling in the order of mΩ · cm, is comparable to some of the iron-based pnictides^21^.

In order to extract the magnetic contribution from the Ce 4*f*-electrons, ${\rho }_{mag}$ is introduced by subtracting the corresponding resistivity of LaNi_2_As_2_ which is presented in Fig. 3(a). The temperature dependence of ${\rho }_{mag}(T)$, normalized by the value at *T* = 300 K, is plotted in the left axis in Fig. 3(d) (for *x* ≥ 0.6). The ${\rho }_{xx}(T)$ curves show different local maxima, *T*_*CEF*_ and *T*_*coh*_; their values are determined from the intercrossing of two dashed lines as indicated in Fig. 3(a,d). Roughly speaking, *T*_*CEF*_ corresponds to the Kondo scattering on the ground state and the excited CEF levels, while *T*_*coh*_ reflects the onset of a coherent state. *T*_*CEF*_ depends weakly on *x*, and decreases slightly in the P-rich side only. Because of the semimetal behavior for *x* ≤ 0.5, *T*_*coh*_ develops when *x* ≥ 0.6 in the P-rich regime where the magnetic moments are fully screened. In our measurements, *T*_*CEF*_ and *T*_*coh*_ merge at *x* ≈ 0.65, after that an intermediate valence regime is entered where the Kondo coherence is expected to become larger than the CEF splitting energy. We plot the coefficient *A* vs. *T*_*coh*_ in a log-log scale on the right panel of Fig. 3(d). The curve follows the expected relationship of $A\propto {({T}_{coh})}^{-2}$ for 0.7 ≤ *x* ≤ 0.9 (see the dashed-dotted-dotted line in Fig. 3(d)), but deviates significantly as *x* is larger than 0.9, suggesting a valence crossover induced by the P-doping. More evidence, such as x-ray absorption, will be instructive to further characterize the valence state.

For *x* = 0.7, a sudden drop of about 15% in ${\rho }_{xx}(T)$ is presented below *T*′ = 0.51 K, seen in Fig. 3(b). We find that this slight anomaly can be suppressed by applying either a magnetic field of *B* = 0.15 T or a large amount of current of *I* = 4 mA. Traces of such effect are also observed for *x* = 0.6 and 0.8, of which the resistivity starts to decrease though not as strong as that of *x* = 0.7. It might be a signal for the system entering a coherent superconducting phase in the very low temperature region for these samples (*x* = 0.6–0.8). However, this possibility can not be concluded in our present measurements since a superconducting impurity phase can not be fully excluded. Further measurements down to much lower temperatures and high quality single crystals are highly desirable to clarify this anomaly.

### Discussion

**Figure 6:**
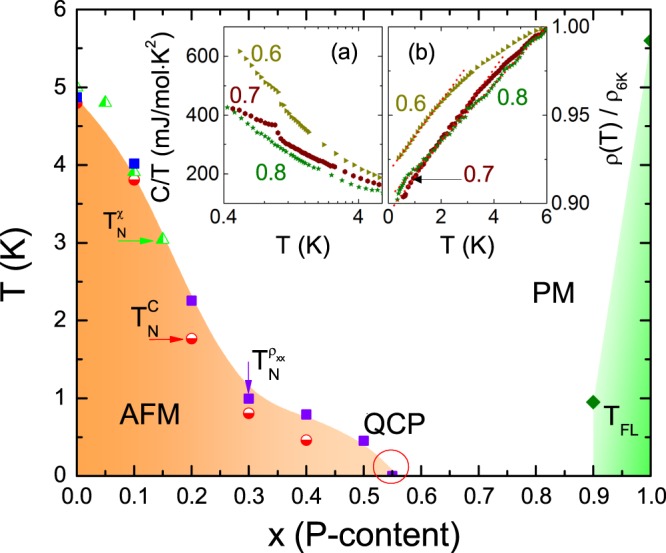
Phase diagram of CeNi_2−*δ*_(As_1−*x*_P_*x*_)_2_ as a function of P concentration *x*. Solid squares, half-filled triangles, and half-filled circles correspond to the AFM transition temperatures from different property measurements: electrical resistivity, magnetic susceptibility and specific heat. Fermi liquid state is recovered below *T*_*FL*_ which is shown as filled diamonds. Around *x*_*c*_ = 0.55, QCP is marked by a circle. Inset (**a** and **b**) respectively show the specific heat divided by temperature, *C*/*T* versus log(*T*), and the normalized electrical resistivity, ${\rho }_{xx}(T)$/${\rho }_{6K}$, below 6 K for *x* = 0.6, 0.7 and 0.8. The dashed lines are linear fits to ${\rho }_{xx}(T)$.

The deduced phase diagram for CeNi_2−*δ*_(As_1−*x*_P_*x*_)_2_ is presented in the main panel of Fig. 6 in terms of P doping concentration *x*. The value of ${T}_{N}^{{\rho }_{xx}}$ is slightly higher than ${T}_{N}^{C}$ from the bulk measurements, especially for samples with 0.2 ≤ *x* ≤ 0.5. Nevertheless, the Néel temperatures determined from different techniques are in reasonable agreement with each other. The isovalent substitution of P for As in CeNi_2−*δ*_(As_1−*x*_P_*x*_)_2_ results in lattice contraction which in turn leads to the enhancement of Kondo coupling *J* between the conduction electrons and the 4*f* moments. Consequently, the long range AFM order is suppressed continuously by the Kondo coupling or *c*-*f* hybridization for *x* ≤ *x*_*c*_. Within the resolution of our measurements, the critical P concentration *x*_*c*_ is close to 0.55 (more precisely, somewhere between 0.5–0.6). Paramagnetic state is entered as *x* is larger than 0.55. The itineracy of Ce 4*f*-electron is enhanced as evidenced from the reduction of the effective moment. Very close to the magnetic instability *x*_*c*_ = 0.55, the specific heat coefficient $C/T$ diverges logarithmically, $C/T\propto -\,\mathrm{log}(T)$, and the electrical resistivity varies linearly, ${\rho }_{xx}\propto T$. Both of them are typical NFL behavior that appears in the vicinity of a QCP. Note that around the QCP, there is no hysteresis loop in ${\rho }_{xx}(T)$ and $C(T)/T$, implying the second-order nature of the quantum phase transition. Since the valence instability is well separated from the fluctuations of the magnetic order parameter, the quantum criticality is of magnetic nature. Slightly above the QCP, at *x* ≈ 0.65, the Kondo temperature and the CEF energy become compatible: *T*_*coh*_ ≈ *T*_*CEF*_. This is reminiscent of the overlap regime in the phase diagrams of Ce122 heavy fermion superconductor family^22–24^.

The P-end parent compound CeNi_2−*δ*_P_2_ shows the typical Fermi liquid behavior: ${\rho }_{xx}\sim A{T}^{2.2}$, and $C/T$ saturates in the low temperature limit with moderately enhanced *γ*_0_ ≈ 209 mJ/mol · K^2^. Note that we actually take the value of *γ* at 0.45 K as *γ*_0_ in the Kadowaki-Woods ratio estimations. The Kadowaki-Woods ratio $A/{\gamma }_{0}^{2}$ is then estimated to be 4.41 × 10^−7^ *μ*Ω · cm(K · mol/mJ)^2^. This value is more comparable to the generalized Kadowaki-Woods relation $A/{\gamma }_{0}^{2}/[N(N-1)/2]$ = 6.7 × 10^−7^ *μ*Ω · cm(K · mol/mJ)^2^ for the Ce-based Kondo lattice compounds with a large orbital degeneracy *N*, rather than the ratio for the standard Fermi liquid with a Kramers doublet ground state^25,26^. These properties suggest that CeNi_2−*δ*_P_2_ has an almost fully degenerate ground state due to the largely enhanced *c*-*f* hybridization compared with the CEF splitting energy. This is further supported by the decreasing tendency: $A/{\gamma }_{0}^{2}$ = 4.59 × 10^−5^ for *x* = 0.6 and $A/{\gamma }_{0}^{2}$ = 1.51 × 10^−5^ *μ*Ω · cm(K · mol/mJ)^2^ for *x* = 0.9.

It is interesting to compare the chemical pressure effect in the series CeNi_2−*δ*_(As_1−*x*_P_*x*_)_2_ with the hydrostatic pressure effect in CeNi_2−*δ*_As_2_ based on the respective phase diagrams. Doping with 55% P leads to a contraction of the unit-cell volume, Δ*V*/*V*_0_ = (V_0_ − V′)/V_0_, of 4.3%. This volume contraction corresponds to that of CeNi_2−*δ*_As_2_ under the critical pressure of *P*_*c*_ = 2.7 GPa^9^. A linear extrapolation towards the 100% P-doping case would suggest that a volume contraction of ≈9.4% corresponds to an estimated pressure of *P* ≈ 6.0 GPa in order to recover the Fermi liquid state. This is over-estimated, since the lattice cell volume does not follow a Vegard’s law between As- and P- rich compounds, but decreases more rapidly beyond 40%. While there is no simple one-to-one quantitative correspondence between the chemical doping and physical pressure experiments, the phase diagrams deduced respectively from chemical doping and hydrostatic pressure show considerable similarities. Both indicate the existence of an AFM QCP that is accompanied by a delayed coherent Kondo screening associated with the low carrier density. The evidence for quantum criticality^27–31^ is clear both at *x*_*c*_ in the present study and *p*_*c*_ in ref. ^9^. Moreover, the similarity in the quantum critical properties, such as the *T*-logarithmic divergence in *C*_*e*_/*T*, at the chemical-pressure-induced *x*_*c*_ here and at the pressure-driven *p*_*c*_ in ref. ^9^ makes a strong case for the intrinsic nature of the quantum criticality.

At the same time, we also identify a key difference. In contrast to the phase diagram of the parent compound CeNi_2−*δ*_As_2_ as a function of hydrostatic pressure^9^, here we find a surprising result in the *T* − *x* phase diagram of CeNi_2−*δ*_(As_1−*x*_P_*x*_)_2_ that the NFL behavior persists over a nonzero range of doping concentration, *x*_*c*_ < *x* < 0.9. As seen as inset (a) to Fig. 6, the specific-heat coefficient of *x* = 0.6–0.8 samples is logarithmically divergent at the lowest measured temperature range, $C/T\propto -\,\mathrm{log}(T)$. The temperature dependence of the electrical resistivity, as shown in the inset (b) to Fig. 6, behaves linearly. The unique NFL behavior is also manifested in the evolution of the power exponent *α*, deduced from the power law fitting described in section D. This exponent *α* increases slightly with increasing *x* from *α* = 0.67 for *x* = 0.55 to 1.18 for *x* = 0.8 but rapidly increases to be larger than 2 for *x* ~ 1.0, signaling the Fermi liquid behavior. We note that in our measurements the Hall coefficient, *R*_*H*_, varies smoothly with *x* across the QCP, as shown as squares in Fig. 2(d). This behavior is in contrast to the related oxypnictides CeNiAs_1−*x*_P_*x*_O in which *R*_*H*_ reveals a drastic change across the QCP^11^. The evolution of *R*_*H*_ in our system may be delayed by the broadened NFL region since *n* increases significantly for *x* = 0.7–0.9. However, we still can not conclude the absence of a drastic change from a smaller 4*f*-localized Fermi surface to a larger 4*f*-itinerant one, since these data are measured at *T* = 2 K. These properties suggest the presence of NFL behavior over a finite zero-temperature region of *x* = 0.6–0.8 in CeNi_2−*δ*_(As_1−*x*_P_*x*_)_2_. This behavior has been reported in the field-tuned transition for Ir-doped^32^ and Ge-doped^33^ YbRh_2_Si_2_.

Our results indicate a NFL phase at dilute carrier limit in CeNi_2−*δ*_(As_1−*x*_P_*x*_)_2_. At exhaustion limit with low conduction electron density, the Kondo energy scale is much lower than the single-ion Kondo temperature *T*_*K*_, resulting in a delayed Fermi-liquid behavior compared with the onset of local Kondo screening^34^. This phenomenon resembles the trajectory “III” in the global phase diagram of the heavy fermion metals (see inset to Fig. 5)^35,36^. Different from the universal SDW-type scenario, i.e., trajectory “II”^27–29^ and the unconventional quantum criticality, i.e., trajectory “I”, which incorporates not only the slow fluctuations of the AFM order parameter but also the energy scale E* associated with the breakup of the Kondo singlet^30,31^, this route describes the quantum phase transition from a Kondo-destroyed antiferromagnetic phase with a small Fermi surface (AF_S_) to a paramagnetic heavy fermion phase with a large Fermi surface (P_L_) through the intermediate Kondo-destroyed paramagnetic phase with a small Fermi surface (P_S_) phase. Note that some NFL features can also be accounted to a random distribution of single-ion Kondo temperatures *T*_*K*_ due to the disorder effect caused by magnetic impurities as investigated in ref. ^37^. The existence of impurities in our samples is reflected from XRD patterns, low-temperature susceptibility, resistivity, and specific heat data. But we note that the peak in the specific heat around 1 K does not vary with the P-doping concentration *x* significantly, implying intrinsic nature of the evolution of the magnetic transition temperature *T*_*N*_ with P-doping. Moreover, the contribution to the total entropy from the impurities is too weak to be identified (data not shown here). We also note that the disorder scenario for the formation of Griffiths-McCoy singularity at low temperatures may be applicable to metallic magnetic systems^38^, while the parent compound CeNi_2−*δ*_As_2_ here is a semimetal-like and CeNi_2−*δ*_(As_1−*x*_P_*x*_)_2_ series show semimetal behavior for *x* ≤ *x*_*c*_. On the other hand, compared with the physical pressure effect study on the single-crystalline sample in ref. ^9^, the similarity between the phase diagrams in ref. ^9^ and this study (Fig. 6) suggests that the disorder effect should not play a major role in the present experiments. Our results set the stage for measurements down to lower temperatures and theoretical analysis on the band structure and Fermi surface topology for further elucidation of the nature of the QCP in this intriguing class of pnictide-based heavy fermion materials.

## Conclusion

In summary, the magnetization, specific heat and transport measurements have been performed on CeNi_2−*δ*_(As_1−*x*_P_*x*_)_2_ (*δ* ≈ 0.07–0.22) system, and a global phase diagram is drawn as a function of P-doping concentration *x*. Evidence of low carrier density is found for 0.1 ≤ *x* ≤ 0.7 from Hall resistivity measurement. The Néel temperature *T*_*N*_ is suppressed continuously upon increasing *x* which is invisible for *x*_*c*_ = 0.55, the QCP. In the vicinity of QCP, the NFL behavior is manifested by $C/T\propto -\,\mathrm{log}(T)$ and ${\rho }_{xx}\propto T$, and a divergent effective carrier mass is evidenced from ${\rho }_{0}$ and *γ*_0_. We find the surprising result that the NFL behavior of the specific heat and electrical resistivity persists over a nonzero range of doping concentration for ${x}_{c} < x < 0.9$; the Fermi liquid behavior is not recovered until *x* ≥ 0.9. We discuss the properties of CeNi_2−*δ*_(As_1−*x*_P_*x*_)_2_ together with those of its 1111 counterpart, CeNi(As_1−*x*_P_*x*_)O, and draw implications for the global phase diagram of the heavy fermion metals. Our present study thus offers a new candidate material for studying the universality classes of quantum criticality, and highlights the effect of the low density of conduction electrons in the nickel-based pnictides.

### Experimental methods

Polycrystalline samples of CeNi_2−*δ*_(As_1−*x*_P_*x*_)_2_ were prepared by solid state reaction method in vacuum. The raw materials are cerium pieces (99.8%), nickel powder (99.999%), and phosphorus powder (98.9%) from Alfa Aesar and arsenic pieces (99.995%) from Aladdin. First, NiAs, NiP, CeAs and CeP were pre-synthesized in a molar ratio of 1:1 in an evacuated quartz tube at 973 K, 973 K, 1323 K and 1173 K, respectively. Second, the initial atomic ratio of 1:1.76:2 were weighted, mixed well, ground, pelletized and installed in Al_2_O_3_ crucibles. Then the crucibles were sealed in evacuated quartz ampoules which were sintered in vacuum at 1323 K to 1570 K for at least 2 days followed by furnace cooling. Finally, the samples were thoroughly ground, cold pressed and annealed in vacuum to improve the homogeneity. Special attentions were paid to the parent compound CeNi_2−*δ*_As_2_ and the non-magnetic counter compound LaNi_2_As_2_: the cold-pressed pellet of the precursors was heated at 1570 K for 2 days without further annealing progress in order to stabilize the low temperature phase with ThCr_2_Si_2_-type structure according to ref. ^39^. It’s noted that since a nickel defect dose not occur for LaNi_2_As_2_, the initial atomic ratio of 1:2:2 is adopted from literature^39^. All the preparation procedures were carried out in an argon protected glove box with the water and oxygen content below 0.1 ppm. The obtained samples are hard and quite stable in the air.

Room temperature powder x-ray diffraction (XRD) measurements of CeNi_2−*δ*_(As_1−*x*_P_*x*_)_2_ were carried out on a PANalytical x-ray diffractometer (Model EMPYREAN) with a monochromatic Cu *K*_*α*1_ radiation. The chemical compositions were verified by energy dispersion x-ray spectrometer (EDS) affiliated to a field emission scanning electron microscope (FEI Model SIRION). The electron beam was focused on a crystalline grain and 3 EDS spectra from different grains were collected to avoid randomicity. In-plane longitudinal electrical resistivity ${\rho }_{xx}(T)$ was measured by the DC four-probe method in a ^3^He cryostat down to 0.3 K (Oxford instrument, model Heliox VL). The magnetic measurements were performed in a magnetic property measurement system (Quantum Design, MPMS-5) with the temperature range of *T* = 2–350 K. The specific heat and transverse Hall resistivity ${\rho }_{xy}(T)$ measurements were carried out in a physical property measurement system (Quantum Design, PPMS-9) down to about 0.5 K.

## Data Availability

The data that support the findings of this study are available from the corresponding author upon reasonable request.
